# Validating human and mouse tissues commonly used in atherosclerosis research with coronary and aortic reference tissue: similarities but profound differences in disease initiation and plaque stability

**DOI:** 10.1016/j.jvssci.2023.100118

**Published:** 2023-07-11

**Authors:** Rogier A. van Dijk, Robert Kleemann, Alexander F. Schaapherder, Antoon van den Bogaerdt, Ulf Hedin, Ljubica Matic, Jan H.N. Lindeman

**Affiliations:** a Department of Surgery, Leiden University Medical Center, Leiden, The Netherlands; b The Netherlands Organization for Applied Scientific Research (TNO), Department of Metabolic Health Research, TNO Metabolic Health Research, Leiden, The Netherlands; c Multi Tissue Center ETB-BISLIFE, Beverwijk, The Netherlands; d Division of Vascular Surgery, Department of Molecular Medicine and Surgery, Karolinska Institutet, Solna, Sweden

**Keywords:** Animal models, Atherosclerosis, Carotid endarterectomy, Comparison

## Abstract

**Objective:**

Characterization of the atherosclerotic process fully relies on histological evaluation and staging through a consensus grading system. So far, a head-to-head comparison of atherosclerotic process in experimental models and tissue resources commonly applied in atherosclerosis research with the actual human atherosclerotic process is missing.

**Material and Methods:**

Aspects of the atherosclerotic process present in established murine atherosclerosis models and human carotid endarterectomy specimen were systematically graded using the modified American Heart Association histological classification (Virmani classification). Aspects were aligned with the atherosclerotic process observed in human coronary artery and aortic atherosclerosis reference tissues that were available through biobanks based on human tissue/organ donor material.

**Results:**

Apart from absent intraplaque hemorrhages in aortic lesions, the histological characteristics of the different stages of human coronary and aortic atherosclerosis are similar. Carotid endarterectomy samples all represent end-stage “fibrous calcified plaque” lesions, although secondary, progressive, and vulnerable lesions with gross morphologies similar to coronary/aortic lesions occasionally present along the primary lesions. For the murine lesions, clear histological parallels were observed for the intermediate lesion types (“pathological intimal thickening,” and “early fibroatheroma”). However, none of the murine lesions studied progressed to an equivalent of late fibroatheroma or beyond. Notable contrasts were observed for disease initiation: whereas disease initiation in humans is characterized by a mesenchymal cell influx in the intima, the earliest murine lesions are exclusively intimal, with subendothelial accumulation foam cells. A mesenchymal (and medial) response are absent. In fact, it is concluded that the stage of “adaptive intimal thickening” is absent in all mouse models included in this study.

**Conclusions:**

The Virmani classification for coronary atherosclerosis can be applied for systematically grading experimental and clinical atherosclerosis. Application of this histological grading tool shows clear parallels for intermediate human and murine atherosclerotic lesions. However, clear contrasts are observed for disease initiation, and late stage atherosclerotic lesions. Carotid endarterectomy all represent end-stage fibrous calcified plaque lesions, although secondary earlier lesions may present in a subset of samples.

## Clinical Relevance

While murine models and (carotid) endarterectomy samples respectively experimental and clinical representatives of the atherosclerotic process, a head-to-head comparison of atherosclerotic with the actual human atherosclerotic process is missing. A systematically applied the revised American Heart Association (Virmani) classification on murine atherosclerosis models and carotid endarterectomy samples. Lesions present were aligned with coronary and peri-renal aortic reference samples that were obtained during organ/tissue donation. It is concluded that the Virmani classification for coronary atherosclerosis can be applied for systematically grading experimental and clinical atherosclerosis. Application of this histological grading tool shows clear parallels for the intermediate human and murine atherosclerotic lesions. However, major contrasts are observed for the process of disease initiation in humans and mice, and aspects of late stage atherosclerotic remained absent in the mouse models studies. It is concluded that carotid endarterectomy specimen all represent end-stage fibrous calcified plaque lesions, although secondary earlier lesions may occasionally present along the primary lesions.

**Article Highlights**

• **Type of Research:** Histological validation study
• **Key Findings:** A head-to-head comparison of human and murine atherosclerosis implies clear contrasts for disease initiation. Parallels are found for the intermediate stages of the disease, but murine lesions do not progress to stage of late fibroatheroma and beyond.
• **Take Home Message:** Although histological parallels are present for the intermediate stages of atherosclerosis, clear contrasts for disease initiation and advanced disease are observed. These observations may provide a rationale for the compromised translation ability of murine findings.

Atherosclerosis is a complex pathology of the large and medium-sized arteries.^1^^,^^2^ There is broad consensus that progression of atherosclerosis, and its clinical consequences essentially relate to qualitative changes in plaque characteristics.^3^ Indeed, insidious manifestations of the disease, such as a myocardial infarction and ischemic stroke, are thought to relate to abrupt plaque destabilization (rupture), rather than to gradual obstruction caused by increases in plaque volume.^3^^,^^4^

Atherosclerosis is a long-term or even life-long process,^5^ that proceeds through well-defined sequential, morphologically defined stages, up to the point of an instable plaque and plaque rupture.^4^ Plaque rupture may trigger thrombus formation, which in turn can elicit a malicious secondary ischemic event (ie, myocardial infarction or ischemic stroke).^4^ Alternatively, plaque rupture may have a more benign prospect with subsequent plaque healing and consolidation (ie, fibrosis).^4^ Grading of these different stages and aspects in the atherosclerotic process fully relies on histological evaluation and classification based on qualitative plaque characteristics.^6^

The first consensus classification scheme for grading coronary atherosclerosis has been introduced by the American Heart Association (AHA) working group,^7^, ^8^, ^9^, ^10^ and has been applied to describe artery-dependent patterns of atherosclerosis.^11^ However, the classification fails to fully capture the different aspects of plaque destabilization and healing, and was therefore refined by Virmani and coworkers.^12^ Based on observations from autopsy material, they extended the AHA classification to better reflect the sequence of events that characterize the disease process, and the heterogeneity of the advanced stages of atherosclerotic disease.

Insight in the molecular aspects of the atherosclerotic process mainly relies on extrapolation of observations from murine models, and clinically on data derived from surgical specimens (in particular, carotid endarterectomy samples). Remarkably, there is currently no uniform classification system for murine atherosclerosis, and a head-to-head morphological comparison of murine models with human disease is missing.^13^, ^14^, ^15^ Similarly, although surgical specimens are sometimes classified in terms of “stable” or “instable,”^16^^,^^17^ the revised AHA classification has not been applied on surgical specimens, and a comparison of surgical and non-surgical specimens is currently lacking.

To test to what extend the spectrum of atherosclerotic lesions represented in the tissue resources commonly used in atherosclerosis research mimic the ‘natural’ history of atherosclerosis, we systematically applied the revised AHA-classification on coronary and (peri-renal) reference tissues, and on the main tissue resources used in atherosclerosis research. The evaluation demonstrates that the standardized classification scheme for coronaries can be universally applied for grading ‘natural’ (native) and experimental atherosclerosis. It is concluded that the tissue resources commonly used in atherosclerotic research only reflect part of the sequence of events that characterize the atherosclerotic disease process.

## Material and methods

Sample collection and handling was performed in accordance with the guidelines of the Medical and Ethical Committee in Leiden, Netherlands and the code of conduct of the Dutch Federation of Biomedical Scientific Societies (https://elsi.health-ri.nl/sites/elsi/files/Code%20Goed%20Gebruik.pdf).

The material and datasets used or analyzed in the current study are available from the corresponding author on reasonable request.

### Human aortic tissue

Tissue sections were selected from a large tissue collection containing over 500 individual abdominal aortic wall patches that were obtained during liver, kidney, or pancreas transplantation (ie, all material was from cadaveric donors). All patches were harvested from grafts that were eligible for transplantation (ie, all donors met the criteria set by The Eurotransplant Foundation), and due to national regulations, only transplantation relevant data for donation is available. Each tissue block in the bank was sectioned, and one section per block was Movat stained and graded (approximately 4000 aorta segments). Details of this bank, including the age and sex distribution of atherosclerotic lesions, have been described previously.^13^^,^^18^

### Human coronary tissue

Tissue sections were selected from a tissue collection, obtained from a large cohort of Dutch tissue donors that contains over 900 individual left coronary artery segments (CAS) that were isolated during aortic valve preparation.^19^ In short, hearts were retrieved from Dutch post-mortem donors within 24 hours after circulatory arrest and brought to the Heart Valve Department of ETB-BISLIFE. There, the major vessels containing the valves were dissected from the heart and stored for clinical use. After dissection, the aortic valve is isolated by trimming the adjacent tissue according to standard procedures. The left coronary artery segment(s) present in the removed tissue are used in this study. This procedure does not interfere with the pathological analysis of the heart necessary for release of the harvested valves. All donors gave permission for research and met the criteria maintained by the Dutch Transplantation Foundation for tissue donation. Each tissue block in the bank was sectioned, and one section per block was Movat stained and graded (approximate 6000 coronary segments). Details of the material in this bank, including the age and sex distribution of atherosclerotic lesions have been reported earlier.^19^

### Human carotid endarterectomies

Tissue sections were selected from a the BiKE biobank, a large tissue bank containing over 700 individual carotid endarterectomy samples that were collected during surgery for a (a)symptomatic carotid stenosis.^20^ For this study, over 100 specimens from the biobank were Movat stained and graded.

### Tissue samples from preclinical models of atherosclerosis

Hearts and aortic tissues were obtained from an extensive mouse tissue biobank (TNO) containing specimen from different atherosclerotic studies performed in Ldlr−/− mice, ApoE−/− mice, and ApoE∗3Leiden mice. Lesion development in these mice was induced through various dietary interventions as for example described in reference 14 (and references therein). Heart and aortic root were collected at sacrifice, embedded in paraffin and subsequently used for preparation of aortic cross-sections as previously reported.^14^ Material from more than 100 mice with ranging cholesterol exposure was Movat stained and graded.

### Characterization of the lesions and histological definitions

All samples included in this study were formalin-fixed and paraffin-embedded. Four μM sections were cut and Movat pentachrome stained for classification of the lesions in accordance with the modified AHA classification as proposed by Virmani et al.^4^^,^^11^ Movat staining allows a clear visual differentiation of the various constituents of the vessel wall, and of the various aspects relevant for grading atherosclerosis. More specifically, the staining identifies mucins/proteoglycans (blue), collagen (yellow), elastic fibers (black), smooth muscle cells, erythrocytes and fibrinogen (red), and nuclei (purple). Different shades of ochre, turquoise, and green reflect co-localization of varying ratios of collagen and proteoglycans.

**Table:** Morphological classification of aortic and coronary tissue according to the modified American Heart Association (AHA) classification proposed by Virmani et al^11^

Subtype of lesion	Abbreviation	Morphological description	Mouse equivalent morphological description
Normal	N	No signs of intimal thickening and intimal inflammation	No signs of intimal thickening and intimal inflammation
Non-progressive intimal lesions			
Adaptive intimal thickening	AIT	Natural accumulation of SMCs in the absence of lipid and macrophage foam cells	Absent in murine models
Intimal xanthoma	IX	Superficial accumulation of foam cells without a necrotic core or fibrous cap	Up to several layers of foam cells without a necrotic core or fibrous cap
Progressive atherosclerotic lesions			
Pathological intimal thickening	PIT	Plaque rich of SMCs and focal accumulation of extracellular lipids with or without the presence of macrophages	Small extracellular lipid pools with overlying or adjacent located macrophages. Intimal SMCs can be identified.
Early fibroatheroma	EFA	Focal macrophage infiltration into areas of lipid pools with an overlying cap	Larger amounts of extracellular lipid with infiltrating macrophages and cholesterol clefts are visible. The core is shielded from the bloodstream by several layers of SMCs. Variable calcification.
Late fibroatheroma	LFA	Loss of matrix and extensive cellular debris with an overlying fibrous cap. Variable (micro) calcification.	N/A
Vulnerable atherosclerotic lesions			
Thin cap fibroatheroma	TCFA	A thin fibrous cap (<65 μm in coronary artery and <155 μm in the aorta) overlying a large necrotic core. Intraplaque hemorrhage can be present in coronary lesions.	N/A
Plaque rupture	PR	Thin cap fibroatheroma with cap disruption with a luminal thrombus communicating with the necrotic core.	N/A
Stabilizing lesions			
Healing rupture	HR	Healed lesion composed of SMCs, proteoglycans, and collagen with or without an underlying disrupted fibrous cap. With or without calcifications.	N/A
Fibrotic calcified plaque	FCP	A fibrous lesion with large amounts of calcification without an underlying necrotic core.	N/A

*N/A*, Not applicable; *SMC*, smooth muscle cell.

A selected number of samples were stained for collagen (Sirius red-picrine method), proteoglycans (Alcian Blue), or immunohistochemical double labeling for CD68 (PA5-78,996; Invitrogen) and α-smooth muscle cell actin (Ab32575; Abcam) using sequential single-labeling immunohistochemistry. Double labeling was achieved by a second heat-induced antigen retrieval after the first chromogen staining in order to inactivate the previous signal and Vulcan red (BioCare Medical) and DAB for visualization. Double-stained slides were not counterstained. Specific antibodies and immunohistochemistry images were captured by means of a digital microscope (Philips IntelliSite Pathology Solution Ultra-Fast Scanner; Philips Eindhoven).

Lesion classification was performed by two independent observers with no knowledge of the patient characteristics of the aortic or coronary tissue. A detailed description of plaque characterization and morphological analysis for aortic and coronary lesions has been provided earlier.^4^^,^^12^^,^^13^^,^^19^ Lesions were classified according to the modified AHA-classification^4^^,^^11^ as adaptive intimal thickening (AIT), intimal xanthoma (IX), pathological intimal thickening (PIT), early (EFA) and late fibroatheroma (LFA), thin cap fibroatheroma (TCFA), acute plaque rupture (PR), healed rupture (HR), or fibrotic calcific plaque (FCP) (Table).

## Results

**Fig 1:**
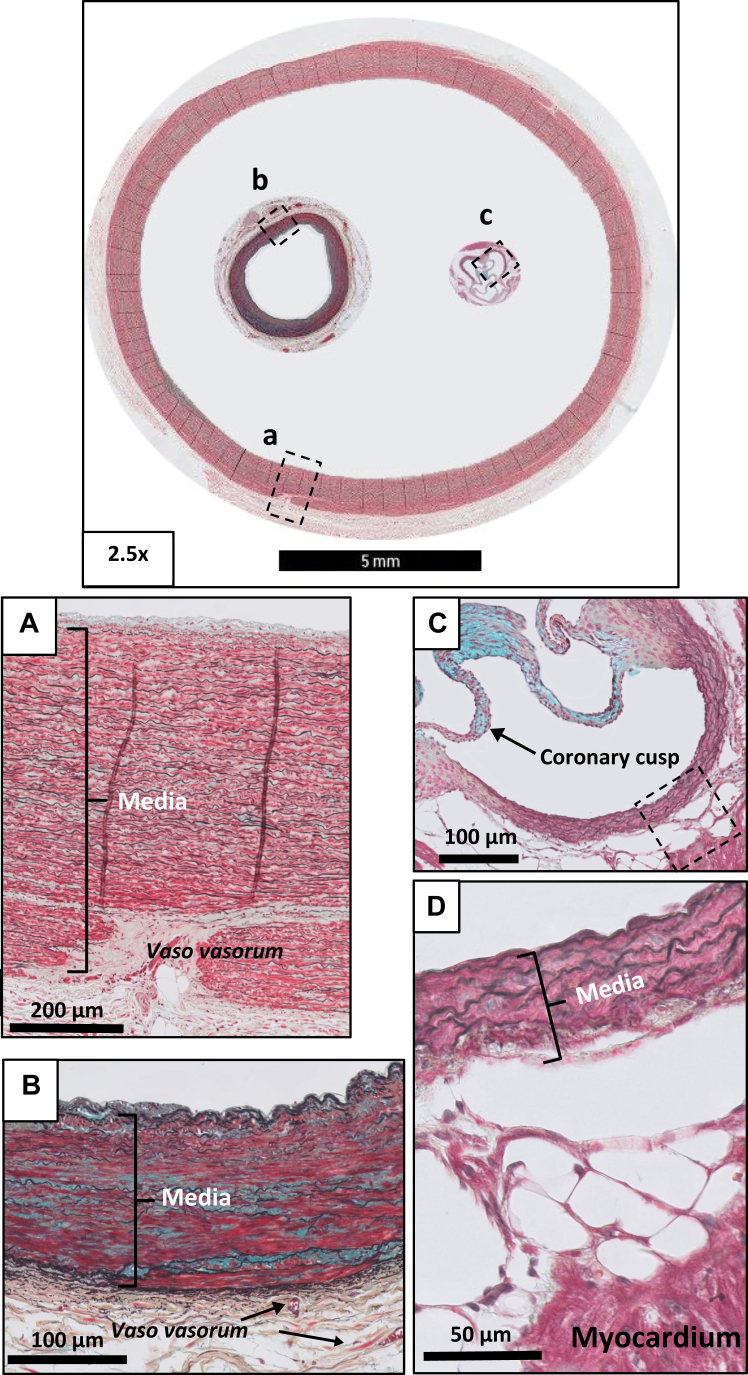
Movat pentachrome staining of a normal human aorta **(a)**, normal human coronary artery **(b),** and normal aortic root in mice **(c)**. Images of a normal human peri-renal abdominal aorta, a normal human coronary artery, and a sample of a normal aortic root of a mouse all taken at the same 2.5× magnification. This image clearly shows how the various vessels relate to one another. Additional high-resolution images of the various vessels clearly show the concentrically arranged fenestrated elastic laminae in the media of the aorta **(A)** and the coronary artery **(B)**, and, to a lesser extent, in the murine aortic root (**C and D** [detail]). The medio-adventitial border of the human aorta and coronary artery consist of vaso vasorum, whereas, by contrast, near the aortic root of mice only myocardium and fatty cells are identified. Note the minimal intimal thickness in the normal murine aortic root.

The obvious size differences between normal human aorta, coronary artery, and the murine aortic root (ie, the predilection places of atherosclerotic lesion formation) are illustrated in Fig 1. The morphology of the intima (composed of endothelial cells with sometimes focal thickened segments of extracellular matrix), is essentially similar for these three vessels (Fig 1, *A-C*). The Movat stain clearly illustrates the different structures of the muscular coronary artery (inner and external elastic lamina) (Fig 1, *B*) and the aorta (media with multiple parallel elastic fibers (black in the Movat staining Fig 1, *A* and *C*). The gross structures of the aortic and coronary adventitia are almost identical, although differences were found the vaso vasorum networks. Although vasa vasora in the coronary artery remain confined to the adventitia, they cross the medial/adventitial border and enter the media in case of the aorta (Fig 1, *A*). The focus in this paper is on the morphological plaque characteristics included in the revised AHA classification, detailed descriptions of the mesenchymal cell dynamics,^21^ and the cellular aspects of the adaptive and innate immune response have been reported earlier.^22^, ^23^, ^24^

Due to the anatomical location of aortic root, the classical adventitia is missing in murine aortic root lesions (Fig 1, *C*). but a classic adventitia is present in the remainder of the murine arterial tree (not shown). Vaso vasorum are absent in the murine arteries.

### Pre-atherosclerotic (reversible) intimal lesions

#### Adaptive intimal thickening and intimal xanthoma

**Fig 2:**
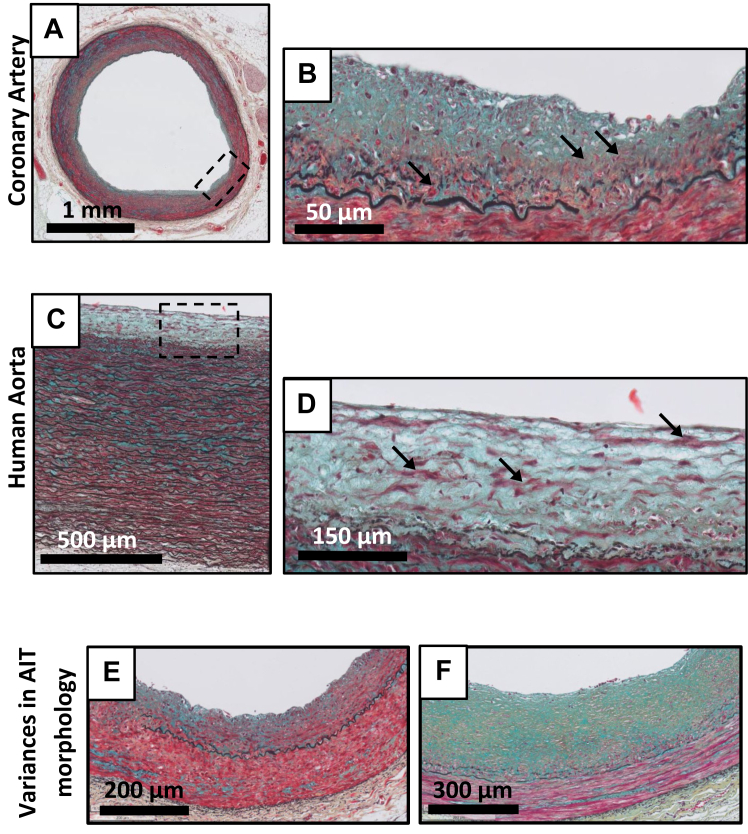
Movat pentachrome staining of adaptive intimal thickening (*AIT*) in the human coronary artery **(A and B)**, the human aorta **(C and D)**. AIT is characterized by accumulating smooth muscle cells (SMCs) (*black arrows* in B and D) in a variable **(E and F)** proteoglycan-rich matrix (*green and turquoise background*). Movat pentachrome staining.

Thickening of the intima, infiltration of smooth muscle cells that appear to transmigrate through a disrupted inner elastic lamina (Fig 2, *A-F*), and deposition of a proteoglycan-rich matrix (Fig 2, *A-F*) are considered the earliest (and reversible) sign of human atherosclerosis.^4^^,^^12^ Microscopically appreciable intracellular lipid deposits, such as foam cells, as well as inflammatory cells are absent. Considerable variation exists in intimal thickness, and mesenchymal cell density in AIT (Fig 2, *E* and *F*). Other than some degree of smooth muscle disorganization in the areas adjacent to the disrupted inner elastic laminae, the media and adventitia appear normal. As illustrated in Fig 2, *A-F*, the histological aspects of this stage are similar for the coronary and the aorta. AIT is not observed in the murine models.

**Fig 3:**
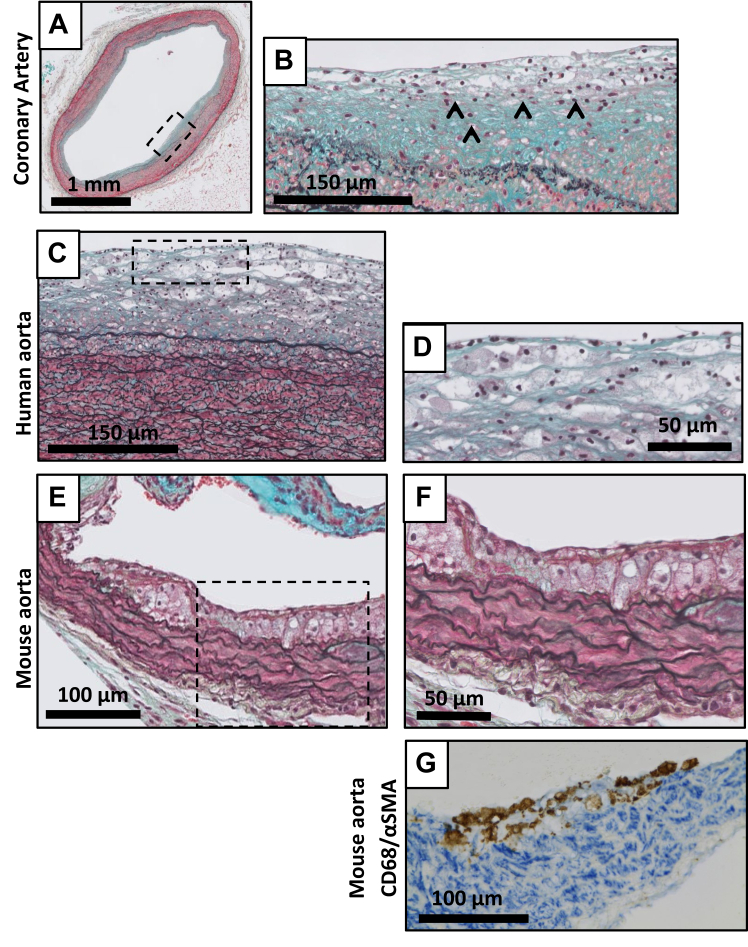
Movat Pentachrome staining of intimal xanthoma (IX) in the human coronary artery **(A, detail B),** the human aorta **(C, detail D)**, and the mouse aorta **(E, details F and G)**. IX are characterized by one or several layers of infiltrating macrophage-derived foam cells (ˆ in **B**) in the intima. CD68 (*brown*)/α-smooth muscle cell (SMC) actin (*blue*) staining shows minimal intralesional SMCs in murine lesions **(G)**.

Transition from AIT to IX in the human lesions is characterized by appearance of intimal foam cells in the smooth muscle cell rich intima (Fig 3, *A-F*). Significant variation is observed in the number of foam cells and the size of the foam cell clusters. By definition (a-cellular) lipid pools are absent, and besides localized smooth muscle disorganization in the inner media directly adjacent to the intima, the media and adventitia are unaffected.

IX is the earliest lesion type observed in the murine models (Fig 3, *E* and *F*). Murine IX lesions are almost exclusively composed of foam cells covered by a layer of endothelial cells. Mesenchymal cells are minimally present, the inner elastic laminae remains intact, and an underlying medial response as observed in humans is not observed in the murine models (Fig 3, *E-G*). Of note, AIT and IX lesions are not observed in carotid endarterectomy specimens.

### Progressive atherosclerotic lesions

#### Pathological intimal thickening and early and late fibroatheroma

**Fig 4:**
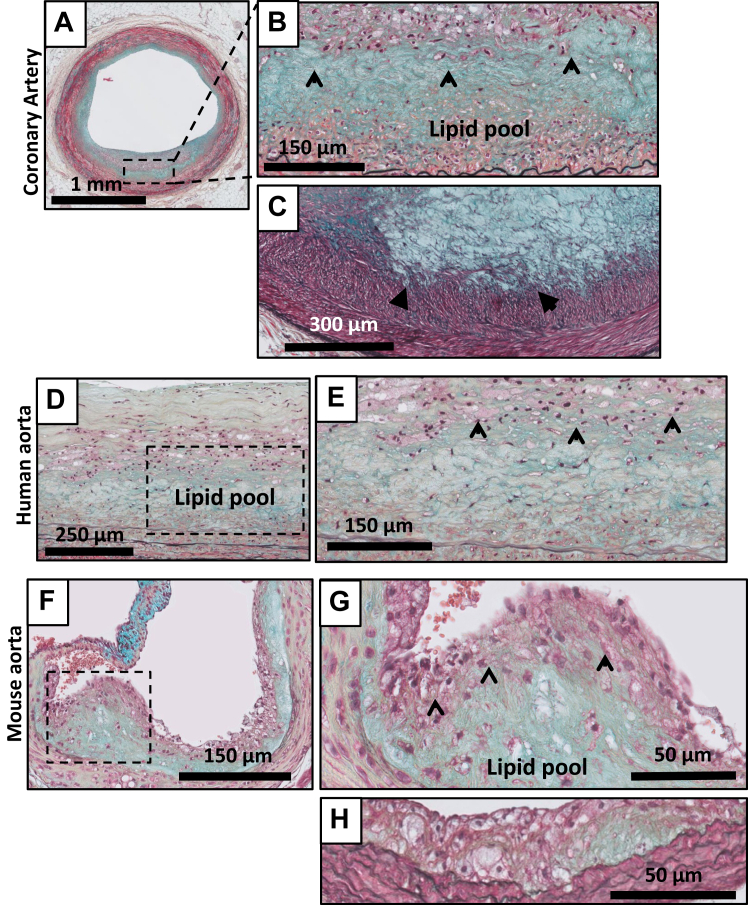
Movat pentachrome staining of pathological intimal thickening (PIT) in the human coronary artery **(A, details B and C),** the human aorta **(D, detail E)**, and the murine aorta **(F, details G and H)**. PIT is characterized by the presence of lipid pools deep within the intima near the intimal medial border with overlying and infiltrating smooth muscle cells (SMCs) in a proteoglycan matrix with or without macrophage infiltration **(B, C, E, and G)**. The morphological appearance is almost identical in all the three vascular beds.

**Fig 5:**
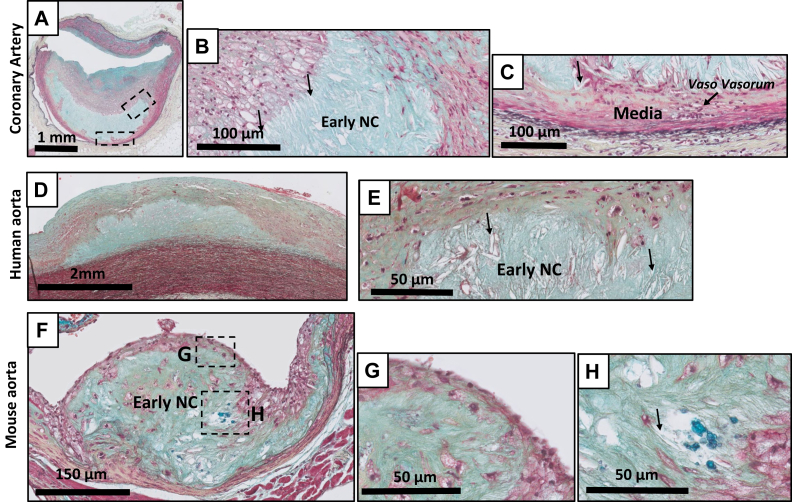
Movat pentachrome staining of early fibroatheroma (EFA) in the human coronary artery **(A-C)**, the human aorta **(D, E)**, and the mouse aorta **(F-H)**. The EFA are characterized by the emergence of delicate cholesterol crystals (*arrows* in **B, E, and H**). Macrophages/foam cells may present adjacent to the early necrotic core (*NC*) **(B)**. Within the coronary artery, the vaso vasorum extends through the media into the intima **(C)**. This is not observed in the human aorta and in mice. Movat pentachrome staining. Calcium deposits (*bright turquoise spots* in **F and H**) can be observed in the murine lesions.

**Fig 6:**
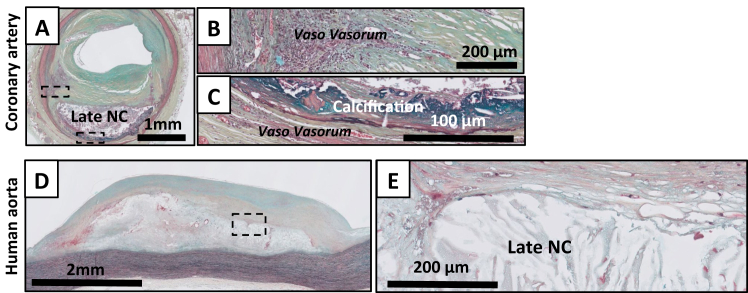
Movat Pentachrome staining of late fibroatheroma (LFA) in the human coronary artery **(A-C)** and the human aorta **(D, E)**. The amorph necrotic core (*NC*) with large cholesterol crystals generally positions deep within the intima near the intimal medial border, areas of calcifications (*dark turquoise staining* in **A and C**) can be present. The lesion is covered by a thick matrix and cell-rich cap. The gross morphologies of the coronary and aortic late NCs are identical, although aortic lesions are generally larger. Unlike the coronary artery, the infiltrating vaso vasorum in the aorta remain limited to the intimo-medial border in the aorta, and intraplaque hemorrhages are absent. LFA are absent in mice.

**Fig 7:**
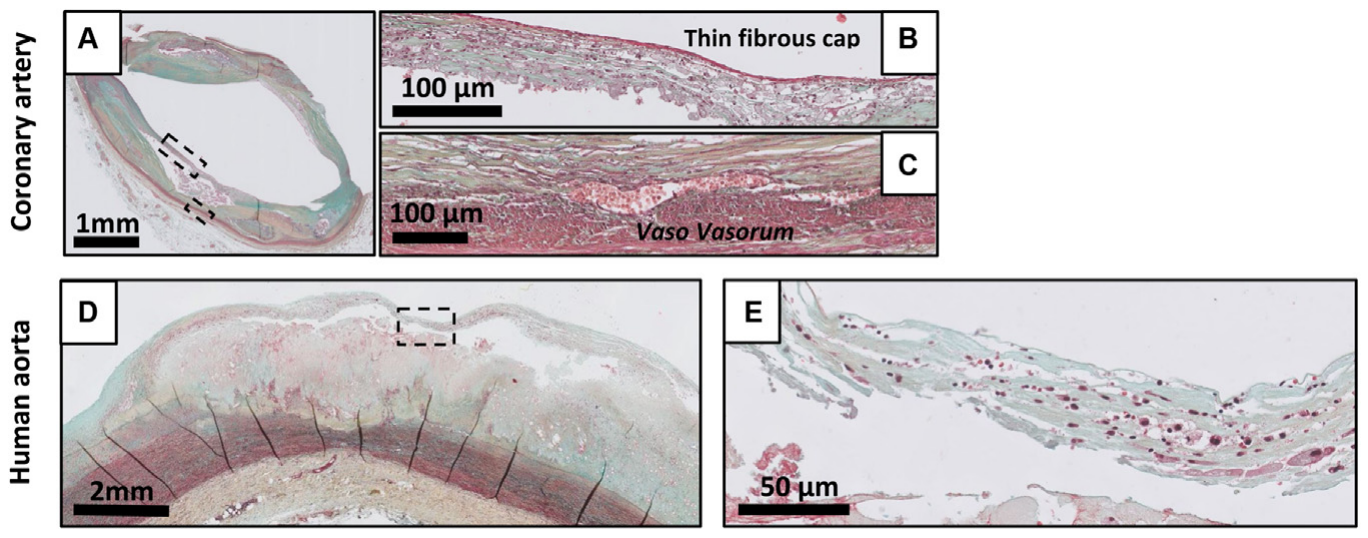
Movat pentachrome staining of thin cap fibroatheroma (TCFA) in the human coronary artery **(A-C)** and the human aorta **(D and E)**. Examples of a TCFA with high resolution images of the thin fibrous cap with excessive macrophage infiltration in the coronary **(B)** and aorta **(E)**, and progressive infiltration of vaso vasorum in the coronary necrotic core. Both coronary and aortic thin cap lesions associate with significant medial thinning and the abundant influx of inflammatory cells in the adventitia **(A and D)**. TCFA are absent in mice.

**Fig 8:**
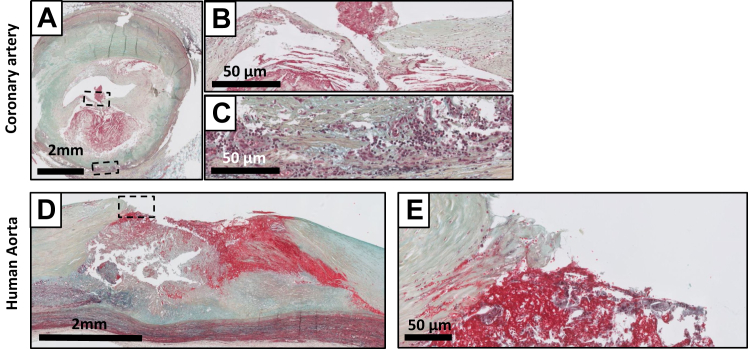
Movat pentachrome staining of plaque ruptures (PRs) in the human coronary artery **(A-C)** and the human aorta **(D, E)**. High-resolution images of PRs with high-resolution images of the thin fibrous cap and the rupture site. Fibrin deposits stain red in the Movat pentachrome staining.

Focal acellular areas consisting of accumulated extracellular lipids within the intimal matrix (ie, lipid pools and later necrotic cores) (Fig 4, Fig 5, Fig 6, Fig 7, Fig 8) characterize progressive lesions. The earliest progressive lesion is referred to as PIT.^4^^,^^12^ This phase is characterized by matrix-rich lipid pools, but absent cholesterol clefts (Fig 4, *A-H*). Lipid pools are mainly located at the medial border zone of the intima and may infiltrate the inner media. The lesions are covered by smooth muscle cells and clusters of macrophages (Fig 4, *B* and *E*). A common phenomenon in the coronary artery, but not in the human and murine aorta, is disruption of the adjacent internal elastic lamina by infiltrating vasa vasora and presence of clusters of basal macrophages/foam cells in the vicinity of these infiltrating vasa vasora (illustrated in a higher magnification image of the coronary artery) (Fig 5, *C*). Coherence of these basal foam cells with lipid pools suggests that these foam cells may contribute to the lipid pool. The morphology of human and murine PIT (Fig 4, *F-H*) is in essence similar, yet infiltrating vasa vasora are absent in murine lesions, and the underlying elastic laminae remain intact.

Transition of PIT to an EFA (Fig 5, *A-H*) is characterized by the emergence of cholesterol clefts (Fig 5, *B*, *E* and *F*) and development of an acellular necrotic core within the existing lipid pools (Fig 5, *B* and *E*).^4^^,^^12^ For the human coronary artery and aorta, the transition associates with an appreciable decrease in proteoglycans within the lipid pools. This, along with progressive accumulation of extracellular lipids, cholesterol crystals, and necrotic debris,^4^^,^^12^ results in the development of an early necrotic core (Fig 5, *B* and *E*). In contrast to the later, more advanced stages, the core still shows a degree of structural organization.

With the exception of intra-plaque hemorrhages (in the coronary generally observed in association with infiltrating vasa vasora), the overall morphological characteristics of the early necrotic core in the human aorta closely resemble that of the coronary artery (Fig 5, *A-E*). Fig 5, *A-G* also illustrates the gross resemblance of the murine and human EFAs: the aspect of the necrotic core of advanced murine lesion bears many similarities with human EFA. However, clear differences are noticed with respect to the tissue response. Murine EFA lack vasa vasora and may contain calcium deposits (Fig 5, *H*). Contrary to human EFA in which the necrotic core is covered by a multi-layered fibrous cap, the core of mouse lesions is shielded from the lumen by a thin layer of smooth muscle cells (SMCs) (see the 400× high resolution images of the EFA in mice: Fig 5, *F* and *G*).

Transition towards an LFA is characterized by further regression of the core,^4^^,^^12^ as it becomes less organized, more translucent, and now presents with large cholesterol clefts (Fig 6, *A-E*). As with EFA, the necrotic core is covered by a thick fibrous cap (ie, a cap with a high collagen content [ochre in the Movat staining]), with varying degrees of infiltration by macrophage and lymphocyte infiltrates.

Importantly, the key characteristics of LFA (late necrotic core with a multi-layer fibrous cap) are absent in the murine models. Although some murine lesions present with larger cholesterol clefts, the level of core-organization remains relatively high, and minimal fibrotic changes occur in the overlying cap.

### Vulnerable atherosclerotic lesions

#### Thin cap fibroatheroma and plaque rupture

Transition from a late progressive lesion (LFA) to a vulnerable lesion (TCFA) is defined by significant thinning of the fibrous cap (Fig 7, *A-E*).^4^^,^^12^^,^^13^ The necrotic core is usually larger; hemorrhage (in coronary lesions) and intraplaque vasa vasora are abundantly present in both coronary and aortic lesions (Fig 7, *C*).

PR is characterized by a disrupted fibrous cap (Fig 8, *A-E*), whereby the overlying thrombus or remnants of thrombus are in continuity with the underlying necrotic core. Most ruptured lesions present with a large necrotic core and a disrupted fibrous cap with infiltrating macrophages and lymphocytes. An -or fragments of- intraluminal thrombus may present at the site of rupture, and wash out of the necrotic core can be occasionally be observed. Vulnerable lesion formation in both the coronary and the aorta associates with a clear adventitial inflammatory response, with formation or tertiary follicles in the adventitia underlying the lesion (Fig 8, *C* and *D*).

TCFA and PR were not identified in mice.

### Stabilizing atherosclerotic lesions

#### Healed ruptures and fibrotic calcific plaques

**Fig 9:**
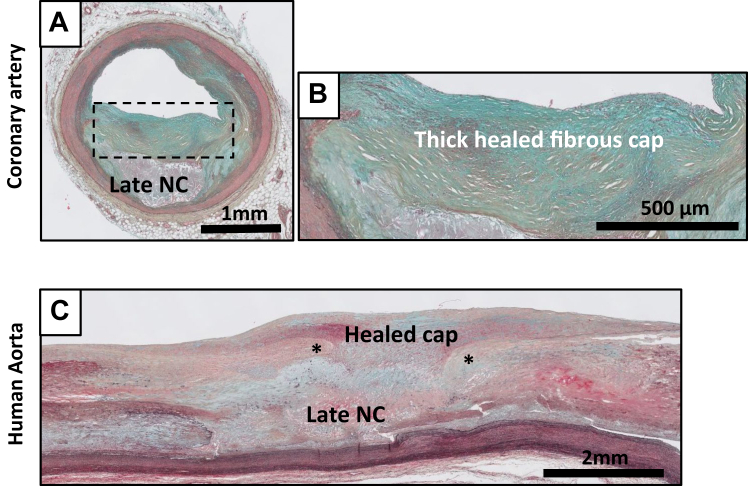
Movat pentachrome staining of healed rupture in the human coronary artery **(A and B)** and the human aorta **(C and D)**. The newly formed, mesenchymal cell and proteoglycan rich (*greenish*) thick fibrous cap shields the remnants of the necrotic core (*NC*) from the lumen. The ∗ denotes the edge of the former ruptured site with remnants of the old cap, and the former, now collapsed necrotic core (late NC).

**Fig 10:**
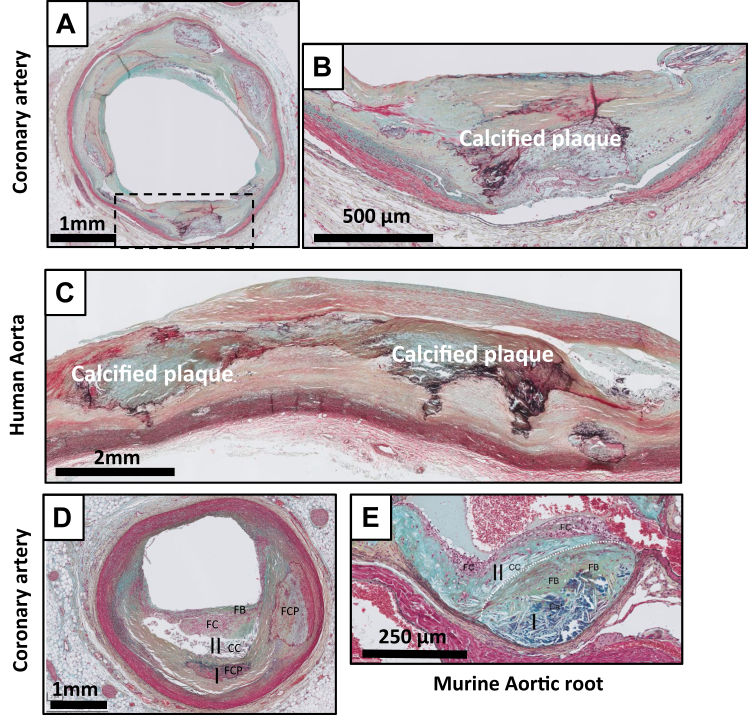
Movat pentachrome staining of fibrotic calcified plaque (FCP) in the human coronary artery **(A and B)** and the human aorta **(C)**, and examples of so called “stacked” atherosclerotic lesions in a human coronary **(D)** and in mice maintained on a prolonged high cholesterol diet **(E). A-C,** Multiple, amorph, matrix-rich calcified areas representing the remnants of the former necrotic core (FCPs) representing the end stage of plaque remodeling. Inflammation and vasa vasora are minimally presented. Note the presence of multiple calcified fibrotic calcified lesions in both examples with an almost circumferential appearance in the coronary. **D**, Consolidated FCP lesion **(I)** with an overlaying LFA lesion **(II)** in a human coronary. **E**, Overlaying early fibroatheroma (EFA) lesions **(I & II)** in ApoE3-Leiden mouse (Ca^+^: calcium deposits). *cc*, cholesterol crystals; *fb*, fibrous cap; *fc*, foam cells; *nc*, necrotic core.

Consolidation of a PR is characterized by a wound healing response in which a disrupted fibrous cap is progressively covered by mesenchymal cells embedded in a proteoglycan cell-rich tissue (green/blue on Movat) (Fig 9, *A-C*).^4^^,^^11^^,^^13^ The matrix within the healed fibrous cap consists of a proteoglycan-rich mass adjacent to, or covering the fibrotic remnants of original cap. Lesions can contain large consolidated areas of calcification with few inflammatory cells and have a small or no necrotic core. The FCPs (Fig 10, *A-C*) reflects consolidation of the healing process. The lesion type presents as a central condensed calcified area at the location of the former necrotic core. Small cholesterol crystals can be present but are absent in the majority of the former necrotic core(s) (Fig 10, *A-D*). Inflammatory cells and tertiary follicles are no longer present in the adventitia, and there appears regression of the pre-existing vasa vasora network. Stacked FCPs (Fig 10, *C*), or earlier lesions on top of FCP (Fig 10, *A*) lesions illustrate the repetitive and chronic character of the human atherosclerotic process in which new lesions can be formed over earlier lesions.

Stacked lesions can be observed in the mice that have been exposed to extreme and prolonged (over 6 months) pro-atherogenic conditions (Fig 10, *D*). Yet, unlike the human situation in which the novel lesions are exclusively observed over consolidated FCP lesions, novel murine lesions overlay intact atherosclerotic lesions (necrotic core), an aspect suggesting absence of human-like PRs in murine models.

### Carotid endarterectomy

**Fig 11:**
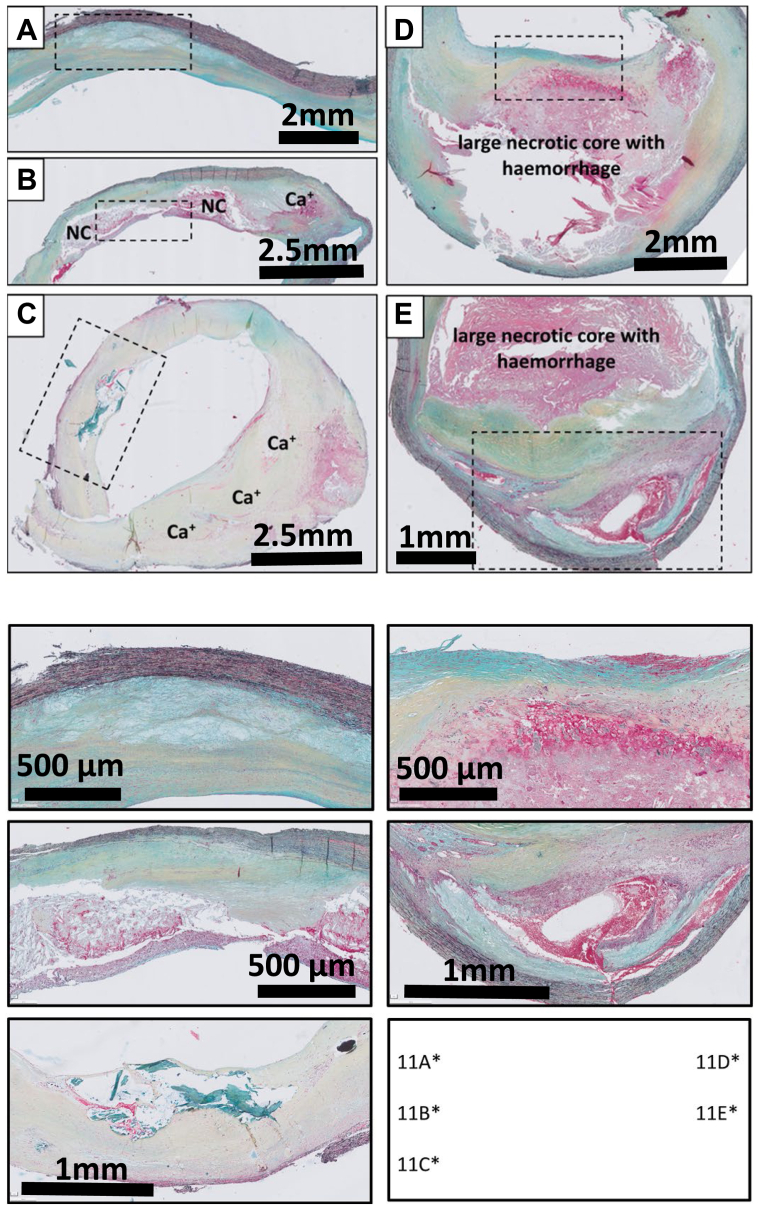
Movat pentachrome stainings of representative examples of carotid endarterectomy samples **(A-E)**. All samples are dominated by the presence of one or multiple calcified fibrotic calcified lesions. Yet, in a subset of samples earlier (secondary) lesions may present next to the primary fibrotic calcified plaque (FCP) lesion. **A,** pathological intimal thickening (PIT); **B and C**, Thin cap atheroma; **D**, Healed rupture; and **E,** recanalization. *NC*, Necrotic core.

Prevailing European guidelines essentially advise carotid endarterectomy for (late stage) symptomatic disease.^25^ This is reflected in the histological aspects of the material included in this study, with all samples studied showing advanced atherosclerotic disease (at least one FCP lesion in the cross-section). However, Fig 11, *A-E* show that adjacent or overlying progressive lesions (PIT and further) are occasionally present along the primary FCP lesion. Morphologic appearances of these earlier lesions are similar to those of the coronaries and aorta, suggesting that the general characteristics of atherosclerotic lesion development in the carotids grosso modo parallel that of the coronaries and the aorta (Fig 4, Fig 5, Fig 6, Fig 7, Fig 8). As the conventional/standard surgical procedure only involves dissection of the intima layer (and occasionally part of the medial layer), information on the other layers (ie, media and adventitia) is intrinsically not available for endarterectomy samples.

### Surrogate measures of plaque stability in mice

**Fig 12:**
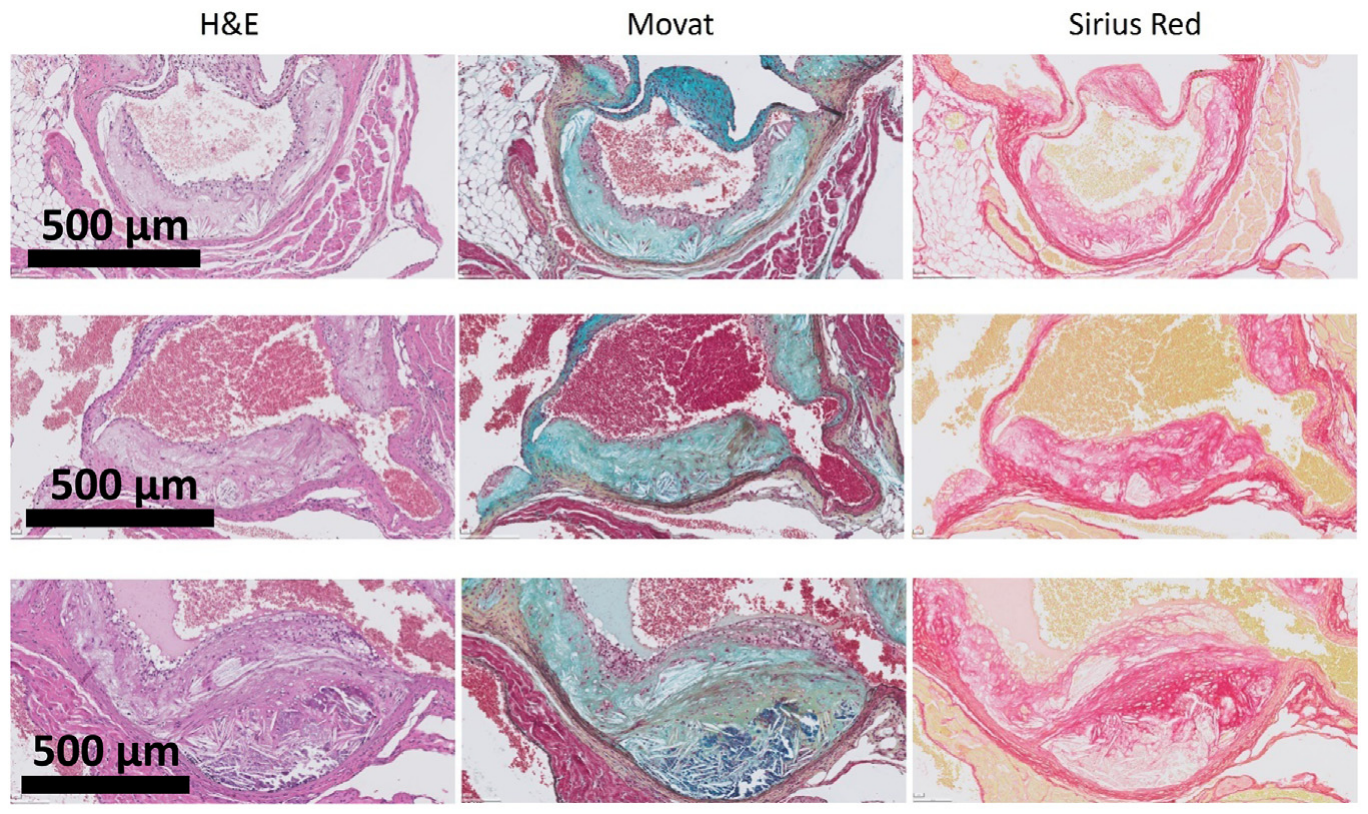
Parallel hematoxylin & eosin (*H&E*), Movat pentachrome, and Sirius red stainings of advanced murine lesions. A classic fibrous collagen rich cap, the hallmark of advanced human lesions, remains absent. Abundant intra-lesional collagen (indicated by the *yellow* collagen [FB] rich area in the Movat stain, and the parallel Sirius red stainings) and calcium deposition can be found in the ‘advanced’ murine early fibroatheroma (EFA) lesions.

Plaque collagen content (lesional collagen) has been brought forward as an estimate of plaque stability in murine models.^26^, ^27^, ^28^ Given the profound differences in the histomorphology of the overlying cap in advanced human and murine atherosclerosis, and the apparent absence of instable lesions in all murine sections studied, we evaluated lesional collagen as surrogate measure of plaque stability for advanced murine atherosclerotic lesions. Fig 12, *A* shows consecutive hematoxylin and eosin (H&E), Movat, and Sirius red staining for ‘advanced’ murine lesions. Movat staining shows absence of a classic fibrous collagen rich cap, the hallmark of advanced human lesions (Fig 7). However, abundant intra-lesional collagen deposition is observed in ‘advanced’ murine EFA lesions. In these lesions, areas of micro-fibrosis present as ochre areas in the Movat stain (and red in the parallel Sirius red staining [Fig 12, *A*]). The two stainings show minimal overlap between plaque collagen content, and cap morphology/collagen content; suggesting that lesional collagen content does not reflect cap characteristics (cap fibrosis). The apparent absence of plaque destabilization at the aortic root is illustrated in Fig 10, *E*. This image shows a typical example of “stacked” atherosclerotic lesions in mice that were maintained on a prolonged high cholesterol diet. In contrast to the human situation in which neo-lesions overlay remodeled (FCP) lesions, all underlying lesions in the murine models were characterized by an intact necrotic core with cholesterol crystals, without indications for (an earlier) hemorrhage (absent fibrinogen).

## Discussion

This systematic histopathologic comparison of human and murine atherosclerosis lesions shows that the established refined AHA (Virmani) classification system for coronary atherosclerosis^4^^,^^12^ is universally applicable for grading of human aortic and carotid, as well as murine atherosclerotic lesions. Based on the histological characteristics, it is concluded that carotid specimens essentially represent end-stage FCP lesions. The analysis further showed that the murine models and conditions included in this evaluation do not progress beyond the stage of EFA. Consequently, aspects of early phases of the atherosclerotic process are not represented in carotid endarterectomy samples, whereas the specific aspects of advanced and vulnerable lesion formation, and successive healing, are not be represented in the material from the experimental murine models included in this analysis.

Staging the successive phases of the atherosclerotic process and assessment of lesion vulnerability fully relies on histopathological evaluation of plaque morphology. The first comprehensive classification scheme provided by the AHA involved an orderly numerical classification embedding six distinct categories.^7^, ^8^, ^9^, ^10^ Unfortunately, this classification fails to capture several important clinical etiologies of coronary thrombus formation that are distinct from plaque rupture, like plaque erosions and calcified nodules.^4^^,^^12^ It further lacks the recognition of precursor lesions that potentially give rise to clinical events, fails to reflect the dynamics of the disease process, and does not acknowledge the process of plaque healing.^4^^,^^12^ Based on their observations and experiences, Virmani et al^4^ refined the AHA classification, resulting in a grading scheme that is believed to better reflect the successive stages of atherosclerotic disease. To be more specific, this descriptive morphological classification incorporates both the various aspects of vulnerable lesion development and plaque destabilization, thereby better mirroring the pathophysiology and presumably the natural history of the disease.^4^^,^^12^ Over the past years, the Virmani classification scheme has been increasingly employed in clinical research and practice, but it is only sporadically employed for the evaluation of surgical specimen or experimental models of disease.^28^^,^^29^

Application of the Virmani classification critically relies on Movat pentachrome staining. Although technically more challenging than conventional H&E stainings, it provides a superior resolution of the matrix components within the plaque.^30^ Only this staining allows for a full appreciation of the changes in matrix composition and tissue morphology characteristic for the different stages of the atherosclerotic process.^4^^,^^11^ In fact, conventional H&E and Sirius Red stainings fail to mirror the dynamics in the proteoglycan-collagen ratios and the emergence of fibrinogen depositions that occur throughout the atherosclerotic process. (Fig 12, *A*). Application of the Virmani classification^4^^,^^12^ on Movat-stained tissue sections performed herein demonstrates that the aspects of included in the classification scheme are universal for native and experimental atherosclerosis; the classification thus allows for a full morphologic alignment of clinical and preclinical lesions, in both elastic and muscular arteries. It is thus concluded that the revised AHA classification can be universally applied for histological positioning of the models and tissue resources commonly used in atherosclerotic research.

Movat staining and subsequent grading of a broad spectrum of human and aortic murine atherosclerotic lesions indicated clear parallels but also discrepancies between human and murine lesions. Although sex-specific differences exist in lesion distribution,^18^^,^^19^ no sex-specific differences were observed for the aspects used for lesion grading in the revised AHA classification. A first notable and consistent finding among all experimental models and conditions included in this analysis is the absence of a primary mesenchymal response in mice. In fact, although the first stages of human atherosclerosis are characterized by disruption of the internal elastic lamina(e) and the apparent transmigration of medial smooth muscle cells^21^ into the intima (adaptive intimal thickening),^4^^,^^12^^,^^13^^,^^31^^,^^32^ intact elastic lamina(e) are observed throughout the murine atherosclerotic process, and the earliest aortic and brachiocephalic lesions in the experimental models universally characterize as a subendothelial accumulation of foam cells with minimal mesenchymal cell involvement (CD68-α SMC actin double staining) (Fig 3, *G*). These observations suggest that the roles of mesenchymal cells^21^^,^^31^^,^^32^ in experimental and clinical atherosclerosis may not fully overlap.

Application of the Virmani classification on murine lesions further shows that the murine aortic lesions studied do not develop the histological characteristics of human vulnerable lesions, and that, based on their histological characteristics, the most advanced murine aortic atherosclerotic lesions classify as EFA. The notion is mainly based on the relatively well-preserved structure of the lipid core and on the small cholesterol crystals in the most advanced lesions observed. A similar morphology is observed for basal (‘buried’) lesions in stacked atherosclerotic lesions that occasionally present in mice maintained under more extreme experimental conditions. This observation sharply contrasts with the human context (Fig 10, *D* and *E*). Stacked atherosclerotic lesions are commonly observed in the coronaries of middle-aged and older individuals,^19^ yet unlike the murine models, the human underlying (basic) lesion(s) universally classify as FCP lesions with a highly condensed and remodeled core that is generally devoid of cholesterol crystals.^19^

A further argument for the notion that the murine lesions evaluated in this study do not progress beyond the EFA is the absence of a multi-layered, fibrous collagen cap that characteristic for the human LFA and TCFA lesions.^4^^,^^12^ One could speculate that, as a consequence of the small anatomical size, the trigger(s) for underlying fibrous cap formation is lacking in murine models (because of the differences in tissue oxygen tension^33^ and in the physical forces exerted on the lesion).

The notion that murine lesions generally do not progress to advanced, vulnerable lesions challenge the use of metric such as plaque collagen content (determined by Sirius Red staining) as a surrogate of plaque (in)stability in murine atherosclerotic lesions.^26^, ^27^, ^28^, ^34^ In fact, a parallel evaluation of Movat and Sirius Red stainings (Fig 12) shows, that for advanced murine lesions, reduced plaque collagen content essentially reflects displacement of ground substance by foam cells and/or cholesterol clefts. Hence, in the context of murine studies, the terminus technicus ‘plaque stability,’^26^, ^27^, ^28^, ^34^ based on intralesional collagen content, bears a risk of misinterpretation.

PRs and intraplaque haemorrhages have been specifically described for murine lesions in the innominate/brachiocephalic artery.^27^, ^28^, ^29^ Unfortunately, brachiocephalic lesions with manifest intraplaque hemorrhages and/or PRs were absent in our biobanks. Consequently, we were unable to perform a direct comparison of these lesions with their human counterparts. Yet, evaluation of the histological images included in references 23-26 suggested involvement of (micro)dissections in the lesion destabilization in this model, an aspect that is extremely rare in our clinical reference samples.^12^^,^^17^

Apart from the earlier described differences in metabolic and inflammatory signatures,^35^, ^36^, ^37^, ^38^ our extensive histological analyses provide a number of further clues why murine lesions might fail to progress towards advanced lesions. Compared with their human equivalent, the advancing lesions in mice are significantly smaller in size. In fact, murine lesions do generally not progress beyond the critical 100 to 150 μm diffusion distance.^33^ As a result, the critical diffusion distance for oxygen and nutrients is not reached in murine tissue. Consequently, an ischemic trigger is missing. An ischemic trigger has been brought forward as the critical driver of plaque neovascularization in clinical atherosclerosis.^39^, ^40^, ^41^ For the coronary, intraplaque hemorrhages related to “leaky” infiltrating vessels are thought to contribute to lesion progression through the accumulation of erythrocyte membrane-derived cholesterol.^42^ Remarkably, leaky vessels and intraplaque hemorrhages were not observed in human aortic atherosclerosis. One could speculate that the absence of intraplaque hemorrhages in stable aortic lesions in humans reflects segmental differences in vasa vasora networks.^42^ A similar topographical dependence may explain the reported contrasts in incident intraplaque hemorrhage and plaque stability between murine aortic and banchiocephalic lesions.

Carotid endarterectomy material constitutes a valuable and rich source of human atherosclerotic tissue, and is commonly applied as human reference in translational studies.^20^^,^^43^ An endarterectomy involves surgical dissection and removal of the intimal layer (and plaque) of a target vessel. Vida infra, the material only represents the intima, although, depending on the dissection plane, fragments of the inner part of the media may present. Consequently, aspects of the vasa vasora network and plaque neovascularization cannot be fully addressed in human carotid endarterectomy samples. Grading a large series of carotid endarterectomy samples in this evaluation identified stabilized FCP lesions as the overarching primary lesion type, although earlier lesions presented occasionally in parallel with the primary lesion. The morphological characteristics of these incidental earlier lesions was similar to those of coronary and aortic lesions, and the revised AHA classification could be adequately applied.^4^^,^^12^^,^^13^

The universal dominance of FCP lesions characterizes human carotid endarterectomy samples as representatives of end-stage atherosclerotic disease.^4^^,^^12^^,^^13^^,^^19^ Consequently, it is concluded that carotid endarterectomy specimens cannot be used as a universal surrogate for studying the atherosclerotic process. Because European guidelines generally restrict carotid endarterectomy to symptomatic lesions, it is likely that observed differences in morphology and composition of the primary FCP lesions (in some reports referred to as “stable” and “instable” lesions)^44^ represent different stages of the healing and consolidation (scar formation) process following the clinical event that triggered the surgery,^45^ rather than that it reflects differences in plaque stability.

These limitations for the primary lesions do obviously not apply to the secondary lesions present in a subset of carotid endarterectomies. Based on their gross morphology and the discriminatory aspects included in the revised AHA classification,^4^^,^^12^ these lesions are fully representative for the progressive atherosclerotic lesions (although non-progressive earliest lesions were not observed in the endarterectomy samples included in our evaluation).

## Limitations

This is an observational study with findings based on histological Movat pentachrome staining of human coronary, aortic, and carotid tissue, and mouse tissue of the aortic root region available in the biobanks used. Consequently, findings in this study should be considered in this context, and it cannot be excluded that, for specific models or conditions not included in this evaluation^46^, ^47^, ^48^ or under specific circumstances, more advanced lesions do develop in mice or other species.^46^ Similarly, decisions for carotid endarterectomy were made on clinical grounds following local guidelines. Therefore, conclusions with respect the translatability of carotid endarterectomy material may vary with the indications for surgery.

Yet, histological reference images provided in this paper and earlier reports^4^^,^^12^^,^^13^^,^^19^ do provide a framework for classification of murine or carotid endarterectomy samples beyond those included in this evaluation.

Reference coronary and aortic tissues were obtained during organ and tissue donation procedures; accordingly, transplantation-specific inclusion criteria apply. Hence, due to the exclusion criteria for donation, material from patients with disease-modifying comorbidities such as uremia^47^ or diabetes^48^ is absent. This aspect may, for example, explain the virtual absence of plaque erosions in our material.

## Author Contributions

Conception and design: RVD, RK, AS, JL

Analysis and interpretation: RVD, RK, JL

Data collection: RVD, RK, AS, AVDB, UH, LM, JL

Writing the article: RVD, JL

Critical revision of the article: RK, AS, AVDB, UH, LM

Final approval of the article: RVD, RK, AS, AVDB, UH, LM, JL

Statistical analysis: Not applicable

Obtained funding: Not applicable

Overall responsibility: JL
